# Self-Propelled Motion Sensitive to the Chemical Structure of Amphiphilic Molecular Layer on an Aqueous Phase

**DOI:** 10.3390/membranes11110885

**Published:** 2021-11-17

**Authors:** Muneyuki Matsuo, Hiromi Hashishita, Satoshi Nakata

**Affiliations:** Department of Mathematical and Life Sciences, Graduate School of Integrated Sciences for Life, Hiroshima University, 1-3-1 Kagamiyama, Higashi-Hiroshima 739-8526, Japan; m211767@hiroshima-u.ac.jp (H.H.); nakatas@hiroshima-u.ac.jp (S.N.)

**Keywords:** self-propelled motion, self-propulsion, *Π*-*A* isotherm, π-π interaction, hydrogen bonding

## Abstract

Two novel amphiphiles, *N*-(3-nitrophenyl)stearamide (MANA) and *N*,*N′*-(4-nitro-1,3-phenylene)distearamide (OPANA), were synthesized by reacting nitroanilines with one or two equivalents of stearic acid. We investigated how the molecular structures of these compounds influenced the characteristics of a self-propelled camphor disk placed on a monolayer of the synthesized amphiphiles. Three types of motion were observed at different surface pressures (*Π*): continuous motion (*Π* < 4 mN m^−1^), deceleration (4 mN ≤ *Π* ≤ 20 mN m^−1^), and no motion (*Π* > 20 mN m^−1^). The speed of the motion of the camphor disks was inversely related to *Π* for both MANA and OPANA at the temperatures tested, when *Π* increased in the respective molecular layers under compression. The spectroscopic evidence from UV-Vis, NMR, and ESI-TOF-MS revealed that the dependence of the speed of the motion on *Π* originates from the intermolecular interactions that are present in the monolayers. This study suggests that it is possible to control the self-propelled motion by manipulating contributing factors at the molecular level.

## 1. Introduction

Several types of self-propelled objects that can move either themselves or other materials over a short distance, such as Janus particles and nanorods, have been reported [1,2,3,4,5]. For these self-propelled objects, the driving force of motion is the difference in the interfacial tension around them [6,7,8]. In these systems, amphiphilic compounds are critical because these compounds can alter the interfacial tension.

The introduction of molecular nonlinearity is an important strategy for producing characteristic features of motion in these systems, such as oscillatory motion and bifurcation of motion. In self-propelled objects driven by an interfacial tension gradient, the nonlinearity of the surface pressure (*Π*) vs. surface area (*A*) isotherm is indicative of the characteristic features of motion. We have previously reported that this induces the reciprocating motion of a camphor disk placed on a molecular layer composed of *N*-stearoyl-*p*-nitroaniline (ANA), which is a molecule with a nonlinear *Π*-*A* isotherm [9,10].

In this study, we synthesized novel ANA-based amphiphilic compounds to examine the effect of the hydrophobic alkyl chains on the nature of camphor disk motion in water. Camphor particles, or boats, have been investigated as self-propelled objects on water because they are easily prepared and exhibit uniform motion for at least 1 h. Here, the difference in the surface tension around the objects is generated by the distribution of camphor molecules that dissolve from the objects [5,7,11]. When a camphor disk was placed on the amphiphilic molecular layer distributed on water, the speed of the disk changed under the compression of *A* according to the characteristics of the *Π*-*A* curve. The results suggest that the motion can be controlled by chemical means, that is, the number of hydrophobic chains and intermolecular forces involved between layers.

## 2. Materials and Methods

### 2.1. Materials

3-Nitroaniline and stearoyl chloride were purchased from Tokyo Chemical Industry Co., Ltd. (Tokyo, Japan). Calcium chloride, dichloromethane, diethyl ester, magnesium sulfate, and sodium chloride were purchased from Nacalai Tesque, Inc. (Kyoto, Japan). (+)-Camphor, chloroform, and triethylamine were purchased from Fujifilm Wako Pure Chemical Corp. (Osaka, Japan). 4-Nitro-1,3-phenylenediamine was purchased from Combi-Blocks (San Diego, CA, USA).

### 2.2. Instrumentation

^1^H-NMR spectra were obtained using a JNM-AL400S/JNE-ECA500 spectrometer (JEOL, Tokyo, Japan). Electrospray ionization time-of-flight mass spectrometry (ESI-TOF MS) measurements were carried out using a LTQ Orbitrap XL mass spectrometer (Thermo Fisher Scientific, Waltham, MA, USA). The surface pressure was monitored using a surface pressure meter (Kyowa Interface Science Co. Ltd., HMB, Saitama, Japan). A camphor disk (diameter: 3 mm, thickness: 1 mm, mass: 5 mg) was prepared using a pellet die set designed for FTIR spectroscopy. The self-propulsion of the camphor disk was recorded with a digital video camera (SONY HDR-CX590, minimum time resolution: 1/30 s). UV-Vis spectra were measured using a UV-Vis spectrophotometer (UV-1650PC, Shimadzu Co., Tokyo, Japan).

### 2.3. Synthesis of N-(3-nitrophenyl) Stearamide (MANA)

MANA was synthesized using the following general procedure: stearoyl chloride (844 μL, 2.5 mmol), 3-nitroaniline (379 mg, 1.1 eq.), and triethylamine (625 μL, 1.5 eq.) were mixed in dichloromethane. The solution was stirred for 24 h at 25 °C. The reaction solution was evaporated under reduced pressure. Subsequently, chloroform was added to the obtained powder, and liquid separation was carried out using hydrochloric acid (1 mM) and saturated brine. The organic phase was dried using magnesium sulfate powder, filtered, and evaporated under reduced pressure. The crude product was recrystallized from chloroform. After filtration, the residue was dried to obtain the product as a white powder in 11% yield. The product was identified as MANA by ^1^H-NMR (Supplementary Figure S1) and APCI-TOF MS measurements. ^1^H-NMR (400 MHz, CDCl_3_) δ = 8.30 (s, 1H), 7.91–7.89 (m, 2H), 7.43 (t, *j* = 8.4 Hz, 1H), 2.34 (t, *j* = 7.6 Hz, 2H), 1.68 (m, 2H), 1.19 (br, 28H), 0.81 (t, *j* = 6.4 Hz, 3H). APCI-TOF MS(MeOH) *m*/*z* = 405.31117 ([M+H]^+^ C_24_H_41_N_2_O_3_ peak appeared at *m*/*z* 405.31061).

### 2.4. Synthesis of N,N′-(4-nitro-1,3-phenylene) Distearamide (OPANA)

OPANA was synthesized using a procedure similar to that used for MANA synthesis, except for the following protocols: stearoyl chloride (1.69 mL, 5 mmol), 4-nitro-1,3-phenylenediamine (383 mg, 0.5 eq.), and triethylamine (1.25 mL, 2 eq.) were mixed in dichloromethane. After liquid separation, the crude product was recrystallized from diethyl ester. After filtration, the residue was dried to obtain the product as a powder in 31% yield. The product was identified as OPANA via ^1^H-NMR (Supplementary Figure S2) and APCI-TOF MS measurements. ^1^H-NMR (400 MHz, CDCl_3_) δ = 8.05 (d, *j* = 9.2 Hz, 1H), 7.64 (s, 1H), 7.24 (s, 1H), 6.34 (d, *j* = 8.8 Hz, 1H), 6.20 (s, 1H), 2.35 (t, *j* = 7.2 Hz, 4H), 1.69 (m, 4H), 1.23 (br, 56H), 0.85 (t, *j* = 6.0 Hz, 6H). APCI-TOF MS(MeOH) *m*/*z* = 686.58303 ([M+H]^+^ C_42_H_76_N_3_O_4_ peak appeared at *m*/*z* 686.58313).

### 2.5. Surface Pressure-Area Isotherm Measurements

The variations in surface pressure with decreasing surface area under isothermal conditions were measured using a surface pressure meter. The trough of the surface pressure meter was filled with a 2 mM aqueous calcium chloride solution. A chloroform solution of MANA or OPANA (42 nmol) was dropped onto the aqueous solution using a micro syringe and incubated for 5 min to evaporate the chloroform, forming a monolayer on top of the aqueous phase. The surface area was decreased from 0.021 to 0.005 m^2^ to measure the dependence of the surface pressure on the surface area. The temperature of the aqueous phase was controlled in a thermostatic chamber.

### 2.6. Monitoring of Self-Propelled Motion of the Camphor Disk

(+)-Camphor disks (3 mm in diameter, 1 mm in thickness, 5 mg in mass) were prepared as self-propelled objects using an FTIR pellet die set. The camphor disk was placed on a monolayer of either MANA or OPANA prepared under the same conditions as for the surface pressure-area isotherm measurements. Forty seconds after the placement, the motion of the camphor disk was recorded using a video camera and analyzed using an image processing program, Image J (National Institutes of Health, Bethesda, MD, USA).

### 2.7. UV-Vis Spectroscopy Measurements

A chloroform solution (250 μL, 12 μM) of MANA or OPANA was added to the CaF_2_ substrate (diameter: 20 mm). The substrate was placed in a desiccator under low pressure to remove chloroform, resulting in a MANA- or OPANA-coated substrate. The UV-Vis spectra of the chloroform solution or the obtained substrate were measured using a UV-Vis spectrometer.

## 3. Results

**Figure 1:**
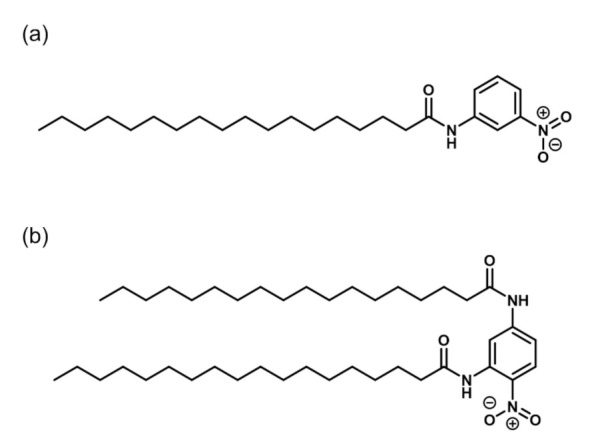
Molecular structures of (**a**) MANA and (**b**) OPANA.

We synthesized novel amphiphiles, *N*-(3-nitrophenyl)stearamide (MANA) and *N,N′*-(4-nitro-1,3-phenylene)distearamide (OPANA), as per the Schotten–Baumann reaction (Figure 1) [9,12,13]. MANA and OPANA were characterized using NMR spectroscopy and mass spectrometry (Supplementary Figures S1 and S2). MANA has a single hydrophobic stearamide chain located at the *meta* position of the aromatic ring, whereas OPANA has two hydrophobic stearamide chains located at the *ortho* and *para* positions.

**Figure 2:**
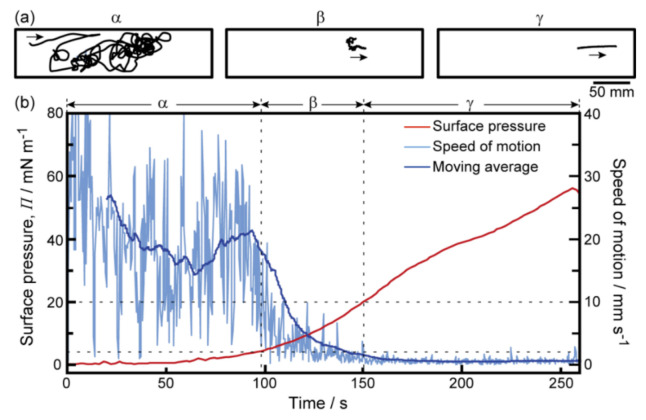
Global motion modes of a camphor disk with a decrease in the surface area of a MANA monolayer containing the camphor disk at 298 K. (**a**) The trajectory of a camphor disk during compression; (**b**) time variation in the surface pressure, *Π* (red line), and the 20 s moving average speed of the camphor disk (blue line) on the monolayer. The pale blue line indicates actual speed of the camphor disk. The reproducibility of these experiments was certified by five trials.

The self-propelled motion of a camphor disk placed on a MANA or OPANA molecular layer was monitored as follows: (1) MANA or OPANA molecules were distributed as a molecular layer on a 2 mM CaCl_2_ aqueous solution. (2) A camphor disk was placed on the MANA or OPANA molecular layer. (3) The camphor disk was monitored under the compression of the molecular layer. The global motion features of a camphor disk were found to change twice: random motion in region α (*Π* < 4 mN m^−1^), globule motion in region β (4 ≤ *Π* ≤ 20 mN m^−1^), and no motion in region γ (*Π* > 20 mN m^−1^) (Figure 2a and Supplementary Movies S1–S4). The mode of motion strongly depended on *Π*, with the threshold between motion and no motion ca. 20 mN m^−1^, similar to previous reports (Figure 2b) [9,11].

**Figure 3:**
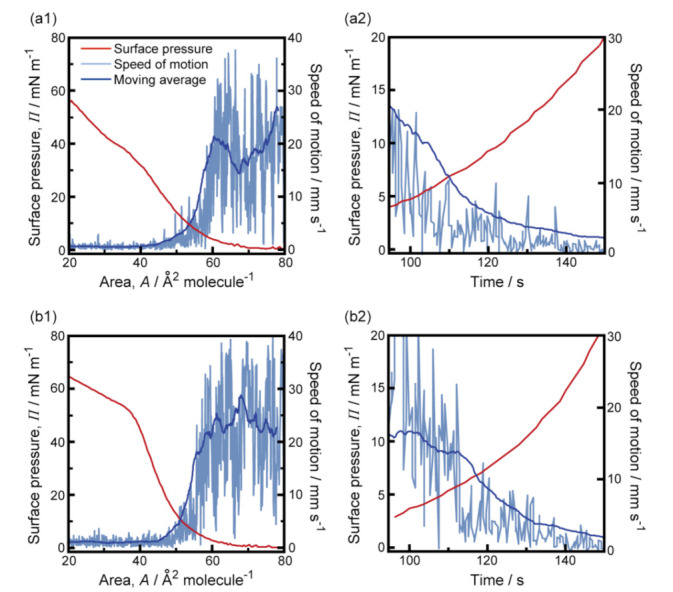
(**a1**,**b1**) Dependence of surface pressure (red lines) and moving average speed of the camphor disk (blue lines) on molecular area of MANA (**a1**) and OPANA (**b1**) at 293 K. Pale blue lines indicate actual speeds of the camphor disk; (**a2**,**b2**) time variations in surface pressure and speed of a camphor disk on the MANA and OPANA molecular layers at 293 K around the region β (4 ≤ *Π* ≤ 20 mN m^−1^) were magnified in (**a2**) and (**b2**), respectively. The reproducibility of these experiments was certified by a minimum of three trials.

Figure 3 shows the experimental results for the simultaneous measurement of the speed of camphor motion and the *Π*-*A* isotherm for the MANA and OPANA molecular layers at 293 K. Around the region β, the speed of camphor motion for OPANA decreased more than that for MANA because *Π* increased more under compression for OPANA than for MANA. The average accelerations estimated from 20 s moving average speeds for MANA and OPANA were −0.26 ± 0.07 and −0.44 ± 0.03 mm s^−2^, respectively. The variations in the local trends of actual motion speeds, such as what is observed between molecular areas of 60 and 80 Å^2^ molecule^−1^ in Figure 3a1, were not generally observed.

**Figure 4:**
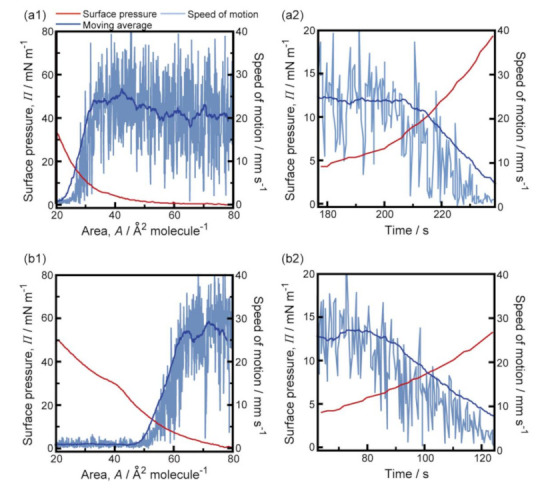
(**a1**,**b1**) Dependence of surface pressure (red lines) and moving average speed of the camphor disk (blue lines) on molecular area of MANA (**a1**) and OPANA (**b1**) at 323 K. Pale blue lines represent actual speeds of the camphor disk; (**a2**,**b2**) time variations of surface pressure and speed of a camphor disk on the MANA (**a2**) and OPANA (**b2**) molecular area at 323 K magnified around the condition of β (4 ≤ *Π* ≤ 20 mN m^−1^). Reproducibility was certified by more than three trials.

Figure 4 shows the results of the simultaneous measurement of the speed of camphor motion and the *Π*-*A* isotherm for MANA or OPANA molecular layers at 323 K. Overall, the values of *Π* were reduced for both MANA and OPANA compared to those at 293 K. In region γ, *Π* increased significantly more under compression for OPANA than for MANA. In region β, the speed of camphor motion for OPANA decreased less than that for MANA because *Π* increased less under compression for OPANA than for MANA. This is a contrast to the results obtained at 293 K. The average accelerations estimated from 20 s moving average speeds for MANA and OPANA were −0.31 ± 0.04 and −0.23 ± 0.04 mm s^−2^, respectively.

**Figure 5:**
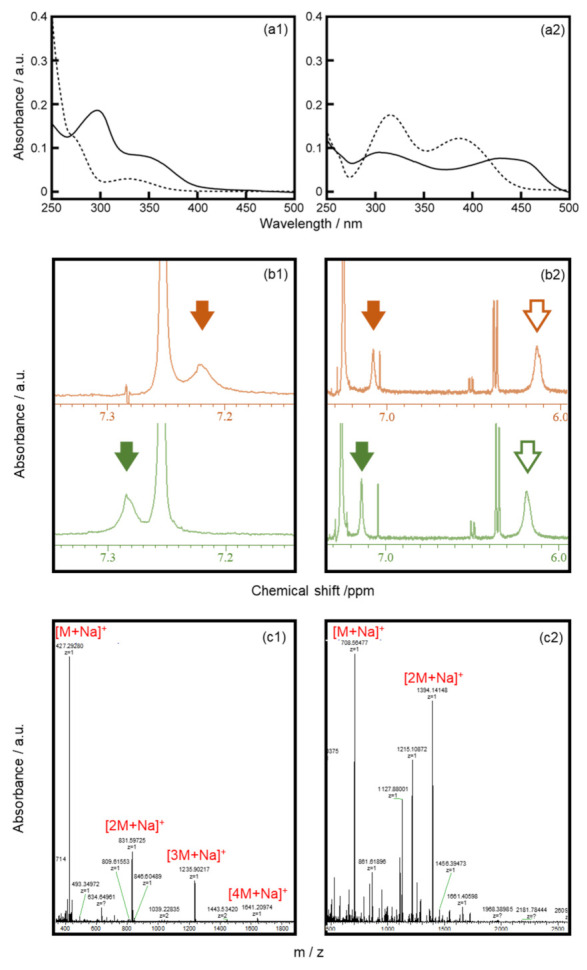
(**a1**,**a2**) UV-Vis spectra for the 12 μM chloroform solution (dotted lines) and solid film (solid lines) of MANA (**a1**) and OPANA (**a2**); (**b1**,**b2**) amide proton chemical shifts in ^1^H-NMR spectra at 293 K (green lines) and 323 K (orange lines) of MANA (**b1**) and OPANA (**b2**); (**c1**,**c2**) ESI-TOF MS spectra measured in positive mode for MANA (**c1**) and OPANA (**c2**). All spectra were obtained from MANA (1) and OPANA (2).

UV-Vis spectroscopy was used to clarify the intermolecular interactions between the OPANA or MANA molecules. Figure 5 shows the UV-Vis absorption spectra of chloroform solutions and solid films on CaF_2_ substrates of MANA (a1) and OPANA (a2). The wavelength of the peak at 390 nm in the OPANA solution spectrum shifted to 430 nm in the solid sample. In contrast, the wavelengths of the two peaks in the MANA solution spectrum at 270 and 330 nm were shifted to 300 and 350 nm in the solid sample, respectively.

To obtain further proof of molecular interactions, ^1^H-NMR (Figure 5b) and ESI-TOF MS (Figure 5c) spectra of MANA and OPANA were measured. Amide proton chemical shifts of MANA moved from 7.29 to 7.22 ppm when the temperature increased from 293 to 323 K. Chemical shifts originating from the amide protons at two different positions of OPANA moved from 6.18 and 7.14 ppm to 6.12 and 7.08 ppm, respectively, when the temperature increased. The proton chemical shifts of hydrogen atoms attached to carbon atoms that are close to the amide bond also moved upfield (Supplementary Figure S3). MANA oligomers ([2M+Na]^+^, [3M+Na]^+^, [4M+Na]^+^) and OPANA dimers ([2M+Na]^+^) were detected as cluster ions in positive mode ESI-TOF MS (Figure 5c).

## 4. Discussion

The data in Figure 2, Figure 3 and Figure 4 suggest that the speed of camphor motion is reflected by the *Π–A* isotherm for both MANA and OPANA: continuous motion occurs at *Π* < 4 mN m^−1^ (region α), a decrease in speed occurs at 4 mN ≤ *Π* ≤ 20 mN m^−1^ (region β), and no motion occurs at *Π* > 20 mN m^−1^ (region γ). This is highlighted by the results in region β, where the deceleration of camphor motion for OPANA is greater than for MANA at 293 K, but is lesser than for MANA at 323 K. This behavior coincided with the change in *Π* in the monolayers under compression. Overall, the higher increase in *Π* under compression for OPANA compared to MANA at both temperatures is due to the existence of one additional hydrophobic group in OPANA. These results suggest that the characteristic camphor motion is explained by the chemical structure of the amphiphilic compound. The reproducibility of the global motion trends under each experimental condition was quite high, despite a lack of reproducibility of the local trends. This may be due to the heterogeneity of the local surfactant distribution, which could be visualized by methods independent of *Π*–*A* measurement, such as ripplon spectroscopy and quasi-elastic laser scattering spectroscopy [14,15].

The peak shifts in the UV-Vis spectra to longer wavelengths (Figure 5a) suggest that MANA and OPANA molecules stack via π-π interactions in the solid state [16,17]. The peak shifts of the amide protons in the ^1^H-NMR spectra of both molecules suggest hydrogen bond formation between molecules for both MANA and OPANA [18,19]. The obtained MS spectra imply that both MANA and OPANA can form multimers through π-π stacking and hydrogen bonding. The weak fluctuation of the camphor motion for OPANA at 293 K can be explained by the π-π stacking interaction between the OPANA molecules. The peak shift of the solid-state UV-Vis spectrum for OPANA was larger than that for MANA, which suggests that the intermolecular interactions within the OPANA molecular layer are stronger than those within the MANA layer.

There is a local minimum in the *Π*-*A* isotherm of ANA, which has a nitro group at the *para* position [9]. There is no local minimum in the *Π*-*A* isotherm of MANA, which has a nitro group at the *meta* position. As a result, reciprocating motion and fluctuating motion occur for ANA and MANA, respectively. Under high compressive conditions, the decrease in *Π* with an increase in the temperature of the aqueous phase may be due to the breakage of the hydrogen bonding within the molecular layers. Under low compressive conditions, an increase in *Π* with an increase in temperature was observed for OPANA but not for MANA. This is consistent with the fact that an increase in the number of alkyl chains suppressed hydrogen bond formation and resulted in the stability of camphor nature, as shown in Figure 3b1 and Figure 4b1.

In vivo, abnormalities in the mobility of membrane proteins in cellular membranes have been shown to induce diseases [20,21]. This work suggests that not only the phase state of the membrane, which has been the focus of much attention in previous works [22,23,24], but also the pressure generated within the membrane, is responsible for abnormal protein motility. The numerical model of surface pressure-dependent camphor disk motions shown in this work, combined with molecular structural information, would be a useful tool for elucidating the influence of surface pressure on membrane protein activity. The homeostasis of membrane protein motility may also be achieved by enzymes such as acetylase and deacetylase, which control the number of alkyl chains for lipids and protein motility.

## 5. Conclusions

In this study, we synthesized two amphiphilic compounds, MANA and OPANA, to investigate the effect of molecular structure on the characteristic features of the motion of a camphor disk on top of a monolayer of these materials. The motion of the camphor disk was dependent on the surface pressure of the monolayers. The features of motion were reflected in the *∏*-*A* isotherms, which depended on the chemical structure and the intermolecular interactions present in the monolayers, notably π-π stacking and hydrogen bonding. This study suggests that characteristic features of self-propelled motion can be controlled from a molecular perspective.

## Data Availability

Not applicable.

## Supplementary Materials

The following are available online at https://www.mdpi.com/article/10.3390/membranes11110885/s1, Figure S1: ^1^H-NMR spectrum of MANA with CDCl_3_, Figure S2: ^1^H-NMR spectrum of OPANA with CDCl_3_, Movie S1: Self-propelled motion of the camphor disk placed on a MANA monolayer at 293 K, Movie S2: Self-propelled motion of the camphor disk placed on a MANA monolayer at 323 K, Movie S3: Self-propelled motion of the camphor disk placed on an OPANA monolayer at 293 K, Movie S4: Self-propelled motion of the camphor disk placed on an OPANA monolayer at 323 K.
