# Supplementary material for: Self-Propelled Motion Sensitive to the Chemical Structure of Amphiphilic Molecular Layer on an Aqueous Phase
Source: Membranes (Basel). 2021 Nov 17;11(11):885. doi: 10.3390/membranes11110885 (PMC8618350; doi:10.3390/membranes11110885)
Supplement: Supplementary file 1 [file membranes-11-00885-s001.zip › Supplementary Files/SI.pdf]

Self-propelled motion sensitive to the chemical structure of amphiphilic molecular layer on an aqueous phase

Muneyuki Matsuo[1,*], Hiromi Hashishita[1], Satoshi Nakata[1]

[1] Department of Mathematical and Life Sciences, Graduate School of Integrated Sciences for Life, Hiroshima University, 1-3-1 Kagamiyama, Higashi-Hiroshima 739-8526, Japan

***** Correspondence: muneyuki@hiroshima-u.ac.jp

Figures

1. ^{1}H NMR spectrum of MANA with $CDCl_3$

2. ^{1}H NMR spectrum of OPANA with $CDCl_3$

Movies

1. Self-propelled motion of the camphor disk placed on a MANA monolayer at 293 K

2. Self-propelled motion of the camphor disk placed on a MANA monolayer at 323 K

3. Self-propelled motion of the camphor disk placed on a OPANA monolayer at 293 K

4. Self-propelled motion of the camphor disk placed on a OPANA monolayer at 323 K

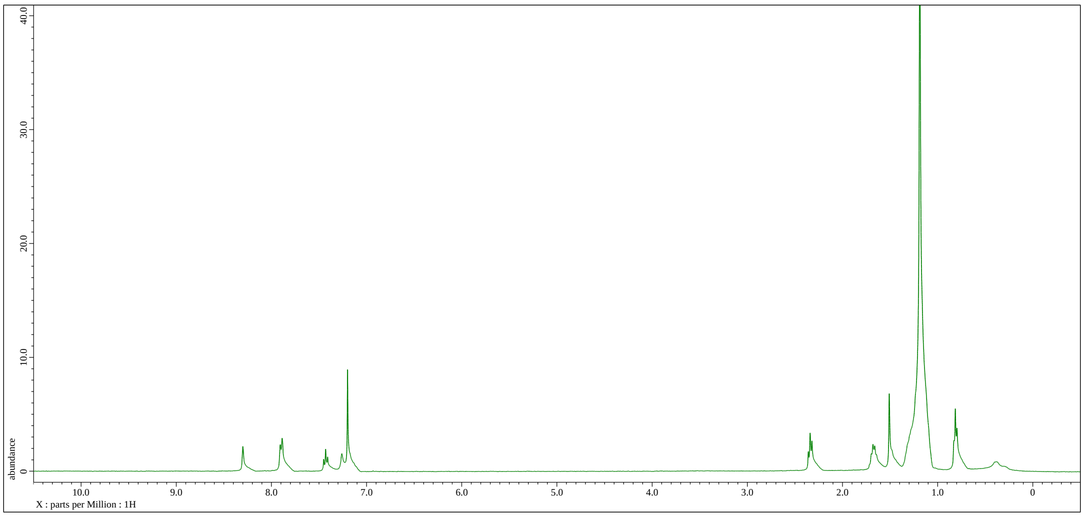

Figure S1. ^{1}H NMR spectra of MANA with $CDCl_3$.

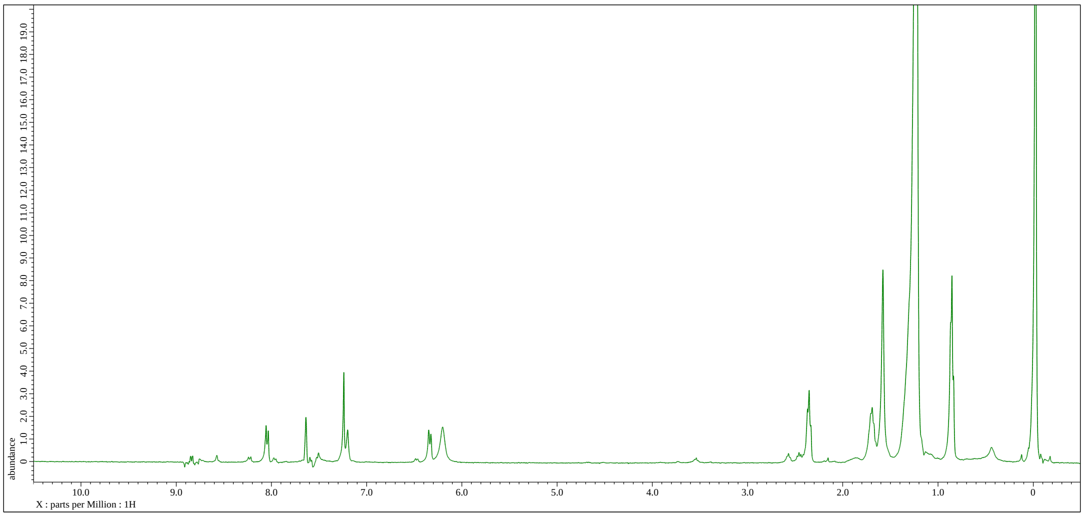

Figure S2. ^{1}H NMR spectra of OPANA with $CDCl_3$.

Movies

Movie S1. Self-propelled motion of the camphor disk placed on a MANA monolayer at 293 K (10× speed, top view).

Movie S2. Self-propelled motion of the camphor disk placed on a MANA monolayer at 323 K (10× speed, top view).

Movie S3. Self-propelled motion of the camphor disk placed on a OPANA monolayer at 293 K (10× speed, top view).

Movie S4. Self-propelled motion of the camphor disk placed on a OPANA monolayer at 323 K (10× speed, top view).
